# The Impact of Carbon Nanofibres on the Interfacial Properties of CFRPs Produced with Sized Carbon Fibres

**DOI:** 10.3390/polym13203457

**Published:** 2021-10-09

**Authors:** Zhenxue Zhang, Xiaoying Li, Simon Jestin, Stefania Termine, Aikaterini-Flora Trompeta, Andreia Araújo, Raquel M. Santos, Costas Charitidis, Hanshan Dong

**Affiliations:** 1 School of Metallurgy and Materials, University of Birmingham, Edgbaston, Birmingham B15 2TT, UK; x.li.1@bham.ac.uk (X.L.); h.dong.20@bham.ac.uk (H.D.); 2 CANOE, Le Centre Technologique Nouvelle Aquitaine Composites & Matériaux Avancés, Bât CHEMINNOV–ENSCBP, 33600 Pessac, France; jestin@plateforme-canoe.com; 3 Research Lab of Advanced, Composites, Nanomaterials and Nanotechnology (R-NanoLab), School of Chemical Engineering, National Technical University of Athens, 10682 Athens, Greece; stermine@chemeng.ntua.gr (S.T.); ktrompeta@chemeng.ntua.gr (A.-F.T.); charitidis@chemeng.ntua.gr (C.C.); 4 Materials and Composite Structures Unit, Institute of Science and Innovation in Mechanical and Industrial Engineering (INEGI), 4000-014 Porto, Portugal; aaraujo@inegi.up.pt (A.A.); rmsantos@inegi.up.pt (R.M.S.); 5 LAETA, Associate Laboratory of Energy, Transports and Aeronautics, 4000-014 Porto, Portugal

**Keywords:** push-out, CNF, carbon fibre-reinforced composite, nanoindentation, contact angle

## Abstract

In this work, different amounts of CNFs were added into a complex formulation to coat the CFs surfaces via sizing in order to enhance the bonding between the fibre and the resin in the CF-reinforced polymer composites. The sized CFs bundles were characterised by SEM and Raman. The nanomechanical properties of the composite materials produced were assessed by the nanoindentation test. The interfacial properties of the fibre and resin were evaluated by a push-out method developed on nanoindentation. The average interfacial shear strength of the fibre/matrix interface could be calculated by the critical load, sheet thickness and fibre diameter. The contact angle measurements and resin spreadability were performed prior to nanoindentation to investigate the wetting properties of the fibre. After the push-out tests, the characterisation via optical microscopy/SEM was carried out to ratify the results. It was found the CFs sizing with CNFs (1 to 10 wt%) could generally increase the interfacial shear strength but it was more cost-effective with a small amount of evenly distributed CNFs on CFs.

## 1. Introduction

Carbon fibre-reinforced polymer (CFRP) composites are light, strong and stiff, and they are widely used in applications requiring a high strength-to-weight ratio, such as aerospace, automotive and civil engineering, and sports equipment, etc. The interfacial properties between the fibre and the resin often significantly influence the performance of the composites, such as the failure mode and fracture toughness. Therefore, much effort is made to improve the bonding between the carbon fibres (CFs) and the matrix via physical or chemical approaches [1]. The surface of the CF is chemically inert; hence, the fibre surface modification to achieve a high specific area, good chemical activity and superior mechanical properties is critical to improve the composite interfacial properties and, ultimately, structural performance. Various techniques [2,3] are used for such purposes: (1) forming reactive functional groups on the surface of the fibres by oxidation or plasma treatment of the CF [4,5,6]; (2) improving the wettability of fibres by using microwaves rather than heating to cure the composite; (3) increasing reactivity and the specific surface area of CF by coating the fibres with nanoparticles (such as carbon nanotubes [7], carbon black, or graphene oxide [8]). Sizing has an easy scalability and is a widely used method in the industry to add particles to the surface of CFs and ultimately to the interface in CFRPs. Carbon nanotubes (CNTs) are widely used in the sizing agents for CFs to increase their bonding with the resin [9], but carbon nanofibres (CNFs) are hardly reported to be used in the sizing agents. CNFs are quasi-one-dimensional carbon materials between carbon nanotubes and CFs, with a diameter in the range from 100 to 500 nm and a length between 0.5 and 200 μm. These types of particles also have a low density, large aspect ratio, large surface area, high modulus and high strength like CNTs [10]. In this paper, the CFs’ surfaces are coated with a very thin layer of a complex formulation, including CNFs via a continuous sizing procedure to examine the suitability of CNFs as a reinforcement agent. This formulation aims to boost the bond between the fibre and the resin in the final composite.

Single-fibre directly loaded and matrix externally-loaded methods are commonly used to determine the parameters of the interfacial interaction between the fibres and matrices [11,12]. Typically, a push-out test is carried out directly on the individual CF in the composite sample that results in the quantitative values of the interfacial shear strength (*IFSS*), which are directly calculated by the critical load, the sheet thickness, and the fibre diameter [13,14].

In this work, the CFs bundle sized with different amounts of CNFs were observed by a scanning electron microscopy (SEM) and characterised by a Raman spectroscopy analysis. The contact angle (CA) of the resin microdroplets on the sized CFs’ monofilaments was used to evaluate their wettability. The sized CFs were also used to produce pre-impregnated materials that were then converted into CFRPs in an autoclave. Nano-indentation was used to measure the nanomechanical properties, such as indentation hardness and a reduced modulus, and to carry out the push-out test. The critical loads of different CFRPs were measured and the *IFSS* was calculated to compare the effect of the addition of CNFs in the sizing agent. Further observations of the CFs by SEM were carried out to validate the push-out results.

## 2. Experimental Details

**Figure 1:**
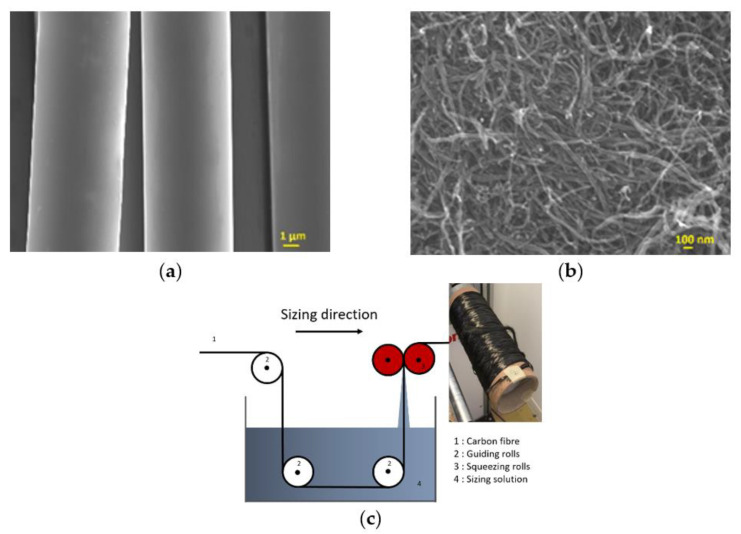
(**a**) T700 CFs, (**b**) CNFs and (**c**) the sizing process.

Bundles of 12,000 filament (12K) CFs T700 SC 31E (Toray carbon fibres Europe) were used in this work which contained an epoxy-compatible sizing agent. A sizing formulation was made of U6-01 (MICHELMAN) composed of a polymer film to bind filaments together, and a polymeric coupling agent to promote fibre–matrix interactions, and thus create strong interfaces between the fibre and matrix resin in the composite. CNFs are not soluble in water due to their inert surfaces; therefore, the non-ionic surface-active agents, Brij S20 (BS20) surfactants, were used to disperse them in aqueous media. The surfactant was a Polyethylene glycol octadecyl ether and had a molecular mass of about 1152 g/mol. The sizing content obtained was up to 2.2 wt.% and the nanofiller concentration in the sizing layer was between 1 and 10 wt.%. As seen in Figure 1a, T700 SC 31 E CFs (T700), with a diameter of approximately 7 μm, were sized with complex formulations containing CNFs (Figure 1b), emulsifiers, anti-static agents and lubricants. A CF spool was first unwound at 5 m/min, passing in a sizing bath, and then it was squeezed into a calendar, dried using infrared (IR) light source combined with hot air, and finally collected onto another bobbin, as shown in Figure 1c. 

**Figure 2:**
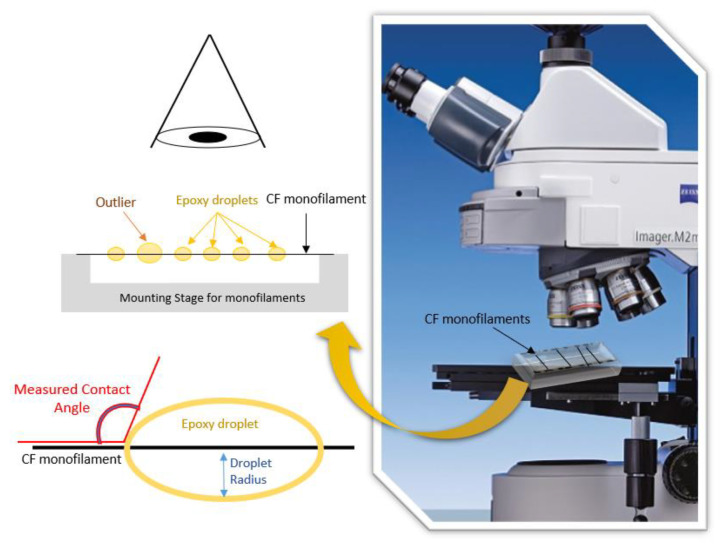
Contact angle measurement on CF monofilaments.

Wettability assessment was performed on the sized CFs by two methods. In the first method, the epoxy droplet spreadability was measured. A bundle of sized CFs was attached to a flat metal substrate, grabbed from both ends, and stretched and glued onto the substrate’s edges. An epoxy droplet was applied on the surface from a 4 mm orifice using gravity (droplet average diameter: 4.5 mm, volume: ~40 mm^3^). The spreading of the droplet on the bundles was recorded by a high-resolution video camera, and captions were taken from deposition time (t_0_) to equivalence time (t_eq_ = 90 s). Three measurements were performed at different points on the surface of the samples, from which the average spreading time and standard deviation were calculated. The measurements took place at room temperature, since a room temperature curing epoxy resin was used, with a low viscosity. For the quantification of the spreading time (by measuring the length of the spreading droplet), the Image J software was used. In the second method, the CA of microdroplets was measured in order to detect differences in the microscale. One monofilament was selected and isolated from the CF bundle and, with the help of scotch tape, it was then attached to a plexiglass holder, under tension. Microdrops (30–200 μm in diameter) of epoxy resin were placed on the monofilaments, using a micro-syringe. For statistical reasons, only microdrops 40–60 μm in diameter were used to measure up to 10 monofilaments. Images for each specimen were captured through optical microscopy using a Zeiss Axio Imager A2.m microscope at the moment of placement, in order to measure the average CA [15]. A schematic representation of the assembly is shown in Figure 2.

The sized CFs were combined with a resin system composed of a diglycidyl ether of bisphenol A (DGEBA, Araldite LY 1556), Aradur 1571, accelerator 1573 and hardener XB 3403 (100:23:5:12 ratio) from Huntsman Advanced Materials^®^ (Bergkamen, Germany), to prepare pre-impregnated materials. Afterwards, the prepreg sheets were cut into sections and converted into CFRP composites with 10 plies, using an autoclave at 120 °C and 3.5 bar for 2 h. The CF volume fraction (V_CF_) of the CFRPs produced was determined according to ASTM D3171 and the results are presented in Table 1.

**Figure 3:**
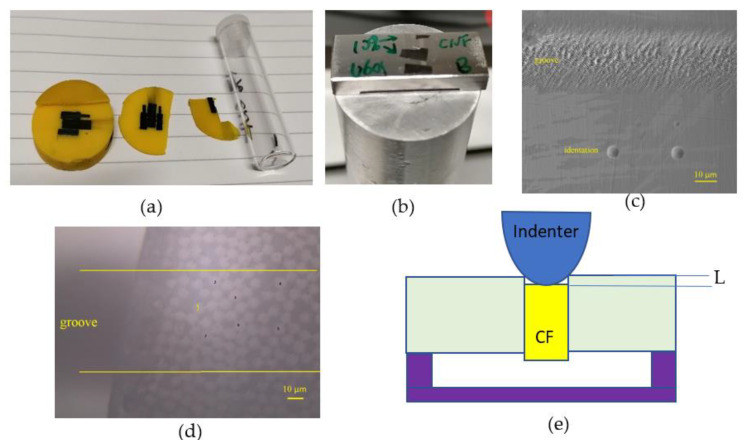
Composite specimen preparation (**a**) pieces cut from mounted samples, (**b**) specimens mounted on the sample holder, (**c**) the round indentations and the groove, (**d**) optical microscopy image of the specimen, and (**e**) schematic of push-out test.

A Micro Materials NanoTest system was used for the nanoindentation and push-out tests. The CFRPs were mounted in bakelite and polished to mirror-like surface for nano-hardness measurements. Afterwards, they were cut to obtain a thin slice with a thickness less than 1 mm. The slice was then broken into a few small pieces by a sharp blade to obtain the CFRP piece (Figure 3a). The polished side of the small piece was attached to an aluminium stud and ground progressively by 1200, 2500 and 4000 grit sandpapers to under 100 µm thick, followed by polishing with colloidal silica suspension on a Struers MD-Chem napped cloth. The thicknesses of the final specimens were between 10–60 µm. The polished specimen was stuck to a rectangle holder with laser machined grooves with a width of 30 µm and a depth of 12 µm, and then glued to the standard cylinder support (Figure 3b). A specially made cone-shaped indenter was used in the push-out test and the round indentation and the laser-machined groove can be seen in Figure 3c. The cone shape ensures an even load and contact with the fibre when compared to a sharp-edged standard Berkovich indenter. The maximum effective depth (L) was about 2.2 µm for a fibre of 7 µm in diameter before the cone contact with the surrounding resin (Figure 3e). The push-out experiments were conducted in an environmental enclosure controlled at 20.0 ± 1.0 °C.

Firstly, in the push-out test, a suitable area on top of the groove (between the two lines) was selected (Figure 3d) and the thickness of the specimen was measured by recording the distance change in the focus from the flat area of the holder to the specimen surface. Secondly, the individual CF, i.e., CF 1, was identified under optical microscopy (Figure 3d). Then, a load was gradually applied to each individual location using the diamond indenter in sequence from an initial load of 10 μN to a maximum peak load at a loading rate of 1 mN/s (Figure 3e). The peak load was greater than the critical load and was decided experimentally for each specimen. The average interfacial shear strength (*IFSS*) at the fibre/matrix interface is provided by [14]:

(1) $$IFSS=\frac{P}{2\pi re}$$

where *P* is the applied critical load, *r* is the fibre radius, and *e* is the sheet thickness. After all the fibres were tested, the fibres in the composite were observed via the onsite microscopy or by a JEOL7000 SEM after unloading from the holder to validate the experiment. The push-out test was carried out at different places with different thicknesses to achieve accurate results.

## 3. Results and Discussion

### 3.1. Carbon Fibres after Sizing CNFs

**Figure 4:**
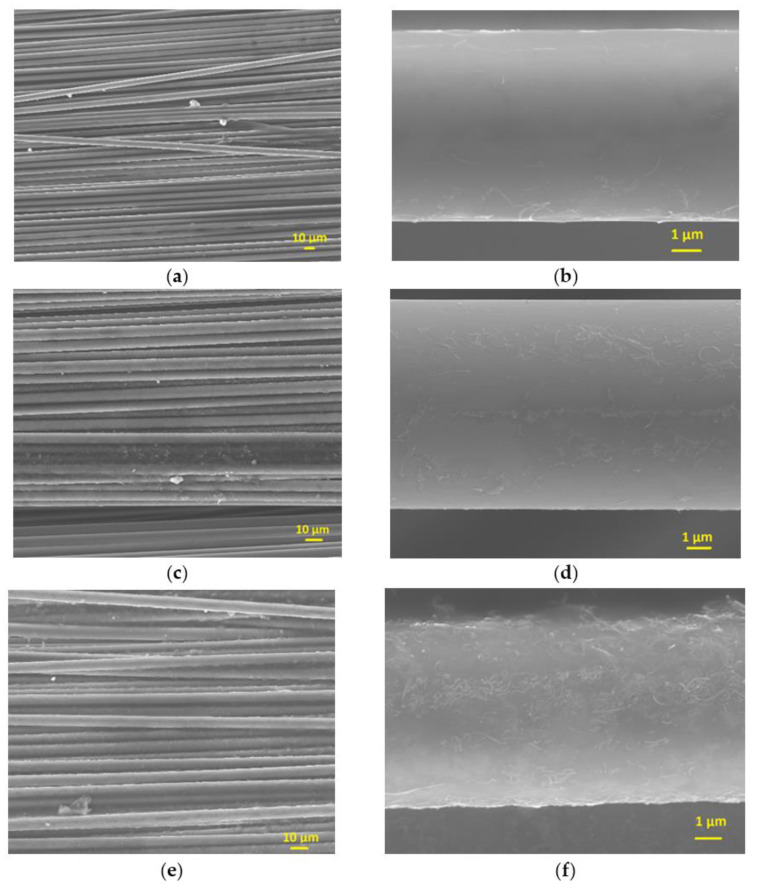
T700 CFs with CNFs in sizing agent (**a**,**b**) 1% CNFs, (**c**,**d**) 5% CNFs (**e**,**f**) 10% CNFs.

**Figure 5:**
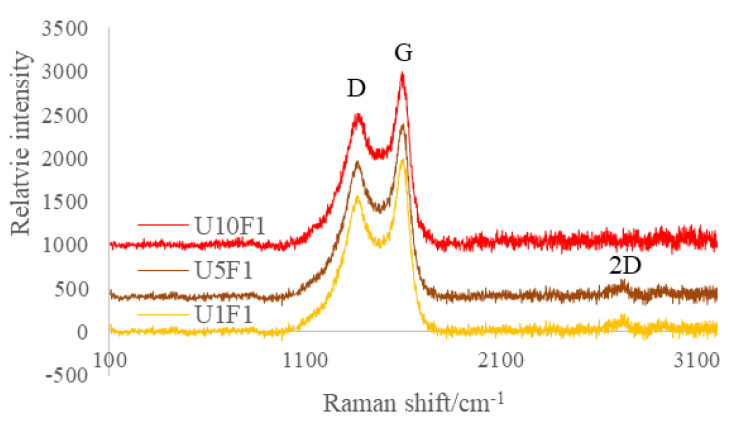
Surface chemistry analyses by Raman on CFs sized with different amount of CNFs.

The unsized CFs’ surface was smooth and clean without any defects (Figure 1a). After CNFs sizing, the surfaces of the CFs were smooth and even (Figure 4a,c,e). For sample U1F1, the CNFs were sparsely distributed on the surface (Figure 4b). When the amount of CNFs in the sizing agents increased, more CNFs were incorporated into the sizing layer of the CFs, as seen in Figure 4d,f. As can be seen in the Raman spectroscopy in Figure 5, G (1595 cm^−1^) and D (1367 cm^−1^) peaks can be identified on the surface of the sized CFs. With the absorbing of 5–10% CNFs on the surface peaks, 2D can also be found at (2722 cm^−1^).

### 3.2. Wettability Assessment of CNF-Sized CFs

**Figure 6:**
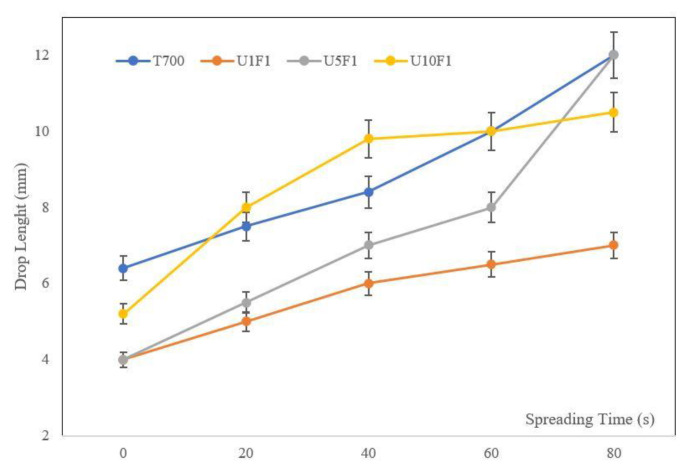
Spreadabillity testing on CNF-based sized CF bundles.

The epoxy droplets spreading on the CNF-based sized bundles of various concentrations are shown in Figure 6. The specimen with 5 wt.% CNFs (U5F1) had a similar behaviour to the commercial sizing until 60 s of spreading, with a slight increase in the deposited droplet at the same time. After 60 s the results showed a sharp increase in spreading. The specimen with a sizing of 10 wt.% CNFs (U10F1) showed the highest rate of spreading for the first 40 s, reaching a plateau afterwards. The lowest CNF concentration sizing (1 wt.% CNFs-U1F1) showed a linear spreading behaviour, with the lowest rate of all the samples tested, indicating that a high affinity was achieved with the epoxy resin.

**Figure 7:**
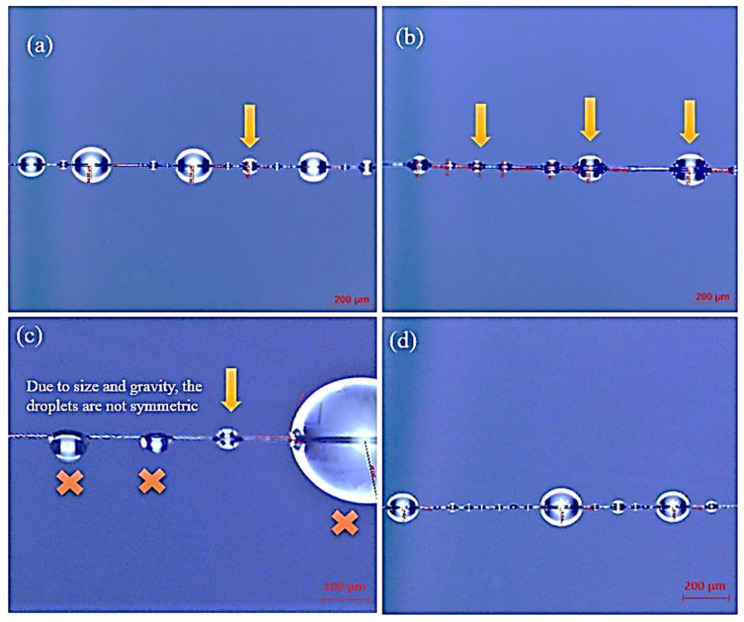
Contact angle measurements of CNF sized fibres with different wt.%: (**a**) T700 (reference), (**b**) 1 wt.% CNF-U1F1, (**c**) 5 wt.% CNF-U5F1 and (**d**) 10 wt.% CNF-U10F1.

According to the spreadability results, it was decided to proceed with single-fibre wettability testing to detect differences in the microscale. The optical images taken on single fibres are shown in Figure 7 and the average CAs are presented in Table 2.

The micro-droplets on the pristine fibre presented a CA of 114.8° whereas, on the sized CFs with 1 wt.% CNFs, significantly larger CAs (163.9°) were measured, indicating the improvement in the wetting properties of the fibres. An improvement in the wettability was also evident for the 10 wt.% CNFs sizing. On the other hand, 5 wt.% CNFs only slightly changed the wetting properties of the fibres in static conditions, which was in accordance with the results of the spreadability testing for the first seconds of measurements.

### 3.3. Nanomechanical Properties of the CFRPs

**Figure 8:**
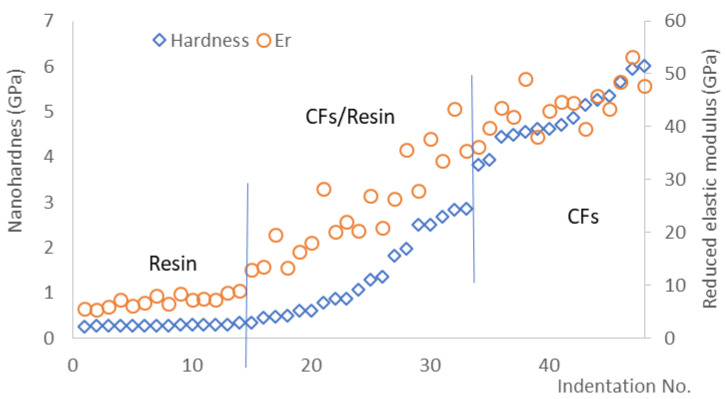
Nano-hardness (H) and reduced elastic modulus (Er) of sized CFs and resin in the composite measured under a load of 5 mN (T700).

A mapping nanoindentation test was carried out to measure the nanomechanical properties of the CFRPs. After the examination of the indentations via microscopy and the corresponding of the indentations to the CFs, the resin, interfaces, hardness and reduced elastic modulus can be calculated; the results of the T700 reinforced composites are shown in Figure 8. CFs have a much higher nano-hardness and reduced modulus than those of the resin. Meanwhile, the hardness and elastic modulus changes gradually at the interface between the CFs and the resin depending on the location of the indentation, which ensures the high-strength carbon fibre reinforces the relatively low-strength resin to obtain the desired properties of the composite materials. There is no significant change in the results measured for the sized CFs with the CNF-reinforced composites; therefore, a more precise method such as the push-out method needs to be used to assess the impact of the addition of CNFs to the interfacial properties.

### 3.4. Push-Out Test

**Figure 9:**
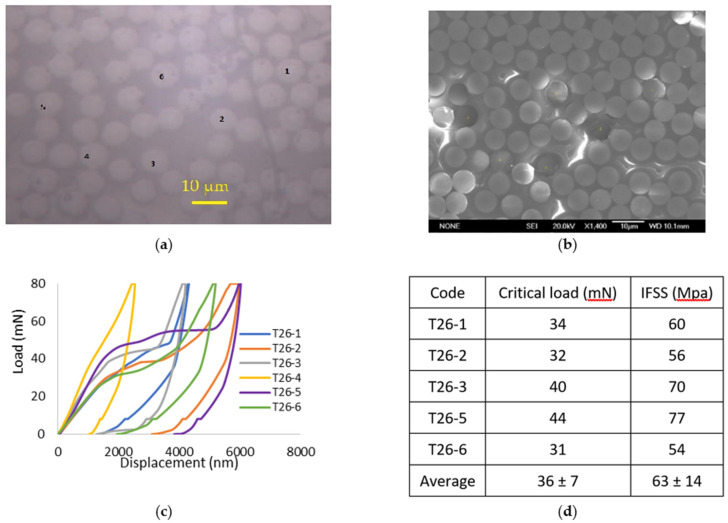
The carbon fibres in a 26 µm (T26) thick T700-31E specimen (**a**) before and (**b**) after push-out test, (**c**) load vs. displacement curves, and (**d**) the critical load and the relevant *IFSS*.

As shown in Figure 9a, the individual CFs were first identified with an on-site microscope, and then the push-out test was carried out on them in sequence. The typical load vs. displacement curves of selected CFs can be seen in Figure 9c. There was a short initial non-linear stage until a conformal contact was reached between the indenter, the specimen and the supporting plate, especially when followed by a linear elastic region (Figure 9c). 

As the load increased to a certain level, a constant load with an increased displacement was reached, which corresponded to the debonding of the fibre, and this was the critical load (P). Observations on the fibres after the push-out test by the on-site optical microscopy and post observation using SEM could validate the results (Figure 9b). For the corresponding curves and indentations analysis, six fibres were used to calculate the interfacial shear strength (Figure 9c). However, fibre 4 strongly interfered with the neighbouring fibres and was not included in the statistics. The corresponding critical load and calculated *IFSS* are shown in Figure 9d for sample T700.

**Figure 10:**
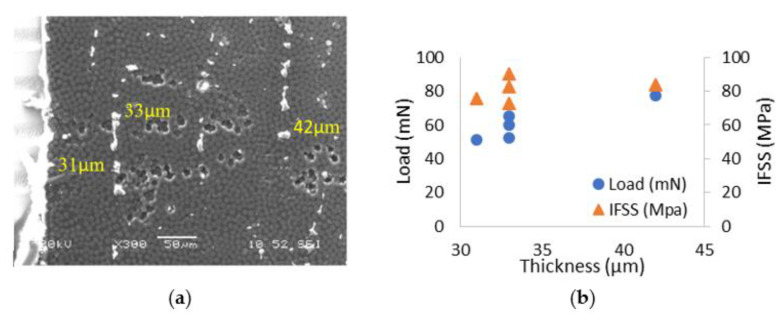
Sample U10F1: (**a**) the area for push-out test with different thicknesses (**b**) the critical load and the relevant *IFSS* with different thicknesses.

For the sample U10F1 (sizing with 10% CNFs), different areas of the samples with varied thicknesses were tested. Generally, the edge of the specimen was thinner, and it became thicker with the move to the centre of the specimen, as seen in Figure 10a. The corresponding critical load and calculated *IFSS* with different thicknesses (31 µm, 33 µm and 42 µm) are shown in Figure 7b. It can be seen that the critical load increases with the thickness of the specimen; however, the *IFSS* only changes slightly with the thickness. Nine tests on a total of 77 CFs were conducted and the average *IFSS* was 72 ± 9 MPa.

**Figure 11:**
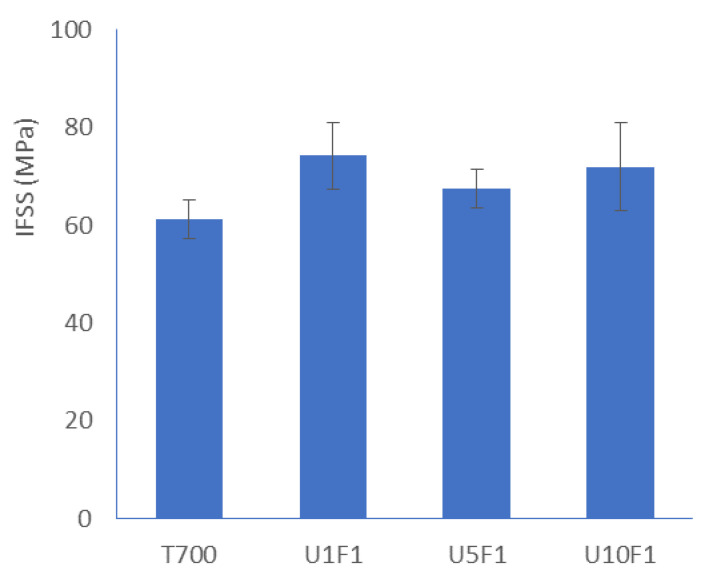
Comparison of the interfacial shear strength with different specimen thickness of the sized carbon fibres in CFRP.

Figure 11 presents a comparison of the *IFSS* results obtained for the reinforced epoxy resin with different-sized CFs. Ten tests were carried out on a total of ninety-four CFs to measure the critical loads at different thickness for the unsized T700 sample, and the average *IFSS* was about 61 ± 4 MPa. The *IFSS* increased for all the samples sized with the addition of CNFs, and U1F1 presented the most significant result of the 74 ± 4 MPa which corresponded to an increase of 21.3% (15 tests on 82 CFs). However, the increased *IFSS* for U5F1 with a 5 wt.% addition of CNFs (7 tests on 54 CFs) was even lower than that of U10F1. This might be because the values were obtained from a thinner sample (<30 µm) which might lead to an easier move from the CFs, and the smaller amount of sizing content (1.4 wt.%) might also contribute to it (Table 1). The other reason might be due to some agglomeration of CNFs on the CFs which negatively affect the bond between the CFs and resin, which has been reported earlier [9].

As previously mentioned, CNFs generally had a larger diameter and length than CNTs; therefore, they were seldom used in sizing agents. However, in this work, it was found that the addition of CNFs in the sizing agent generally had a positive effect on the interfacial strength. The higher amount of added CNFs, aside from not being cost effective, did not lead to a proportional increase in the *IFSS*. Furthermore, high amounts of carbon nano-inclusions tended to cause local agglomeration (Figure 4c–e), which was unfavourable for the improvement of interfacial properties [9].

In short, the sizing agents played a leading role to enforce the compatibility between the CFs and the resin in the composite. The resin droplet remained consistent and took a longer time to spread, which was helpful to prevent interfacial slip. Therefore, the affinity with the matrix was stronger, and the smaller amounts of CNFs could significantly improve the interfacial properties in CFRPs. Furthermore, the agents could cover the CFs smoothly and evenly to avoid agglomeration, which negatively influenced the interfacial strength. In comparison with other carboneous nano-inclusions (Table 3), the sizing with the inclusion of CNFs only showed an approximate increase of 20% in the *IFSS*. Nevertheless, this method normally introduced less damage to the CFs, was simple to operate and easy to scale-up, and could be conveniently incorporated into current production line, making it generally more attractive than other approaches.

## 4. Conclusions

Commercial CFs surfaces were coated with a very thin layer of a complex formulation, including different amounts of CNFs via sizing, which aimed to create strong interfaces between the fibre and the resin. Nano-indentation was used to measure the mechanical properties and assess the interfacial shear strength of the CFs and resin through a push-out test. In the experiment, an individual CF started to slide under a critical load which could be used to calculate the interfacial shear strength (*IFSS*). Although the CNFs had larger dimensions than the CNTs, the addition of CNFs in the sizing agent could generally increase the bond between the CFs and resin, especially with a lower filler amount of 1 wt%. The same also applied for the wettability of the fibres in the microscale, where the 1 wt% of CNFs in the sizing solution presented a high improvement of the wettability. However, sizing with a larger number of CNFs proved to be a less cost-effective approach, since it did not lead to a proportionate increase in the *IFSS*.

## Figures and Tables

**Table 1:** Detail of sizing of T700 CFs with CNFs and CF volume fraction of the respective CFRPs.

Code	Surfactant	% Sizing	% Filler in Sizing	CFRPs V_CF_ (%)
T700	-	-	-	51
U1F1	BS20	2.4	1% CNF	56
U5F1	BS20	1.4	5% CNF	51
U10F1	BS20	1.8	10% CNF	56

**Table 2:** Average measured contact angles and average diameter of the measured micro-droplets.

Code	Droplet Radius (μm)	Contact Angle (deg)	Results
T700	52.6 ± 5.4	114.8 ± 2.3	Reference Value
U1F1	44.3 ± 9.2	163.9 ± 7.8	Highly Improved
U5F1	58.1 ± 3.6	120.3 ±3.6	Slightly improved
U10F1	45.2 ± 7.2	127.3 ± 5.2	Improved

**Table 3:** Carbonaceous materials coated CFs to enhance their *IFSS* [3].

Nano Inclusions	Method	*IFSS* Increase	Potential Issues
CNTs	Dip-coating [16] and Electphoretic deposition [17] CVD [18] Sizing (0.1%CNT) [9]	14–33% 94% 97.6%	Agglomeration, dispersion issue, and solution damage to CFs Thermal degradation and catalyst diffusion Agglomeration
Graphene nanoplates	Microwave enhanced plasma CVD [19]	101.5%	High-temperature treatment (600 °C) and vacuum required
Graphene oxide	Chemical Grafting [20], Electrophoretic deposition and dip coating [16]	11–69.9%	Exposure to an electric field in solution→reduction in CFs strength
Carbon black	CVD at 1000 °C [21]	44%	Thermal degradation and high temperature required.
CNFs	Electro-spinning grafting [22] O-CNFs by Electrophoretic deposition (EPD) [23]	48%	Hybridization and exposure to an electric field in solution could damage the CFs

## Data Availability

Data is contained within this article.
